# OPTIMADE, an API for exchanging materials data

**DOI:** 10.1038/s41597-021-00974-z

**Published:** 2021-08-12

**Authors:** Casper W. Andersen, Rickard Armiento, Evgeny Blokhin, Gareth J. Conduit, Shyam Dwaraknath, Matthew L. Evans, Ádám Fekete, Abhijith Gopakumar, Saulius Gražulis, Andrius Merkys, Fawzi Mohamed, Corey Oses, Giovanni Pizzi, Gian-Marco Rignanese, Markus Scheidgen, Leopold Talirz, Cormac Toher, Donald Winston, Rossella Aversa, Kamal Choudhary, Pauline Colinet, Stefano Curtarolo, Davide Di Stefano, Claudia Draxl, Suleyman Er, Marco Esters, Marco Fornari, Matteo Giantomassi, Marco Govoni, Geoffroy Hautier, Vinay Hegde, Matthew K. Horton, Patrick Huck, Georg Huhs, Jens Hummelshøj, Ankit Kariryaa, Boris Kozinsky, Snehal Kumbhar, Mohan Liu, Nicola Marzari, Andrew J. Morris, Arash A. Mostofi, Kristin A. Persson, Guido Petretto, Thomas Purcell, Francesco Ricci, Frisco Rose, Matthias Scheffler, Daniel Speckhard, Martin Uhrin, Antanas Vaitkus, Pierre Villars, David Waroquiers, Chris Wolverton, Michael Wu, Xiaoyu Yang

**Affiliations:** 1 Theory and Simulation of Materials (THEOS), and National Centre for Computational Design and Discovery of Novel Materials (MARVEL), École Polytechnique Fédérale de Lausanne, 1015 Lausanne, Switzerland; 2 Materials Design and Informatics unit, Department of Physics, Chemistry and Biology, Linköping University, Linköping, Sweden; 3 Tilde Materials Informatics, Straßmannstraße 25, 10249 Berlin, Germany; 4 Materials Platform for Data Science, Sepapaja 6, 15551 Tallinn, Estonia; 5 Theory of Condensed Matter Group, Cavendish Laboratory, 19 J.J. Thomson Avenue, Cambridge, CB3 0HE United Kingdom; 6 Lawrence Berkeley National Laboratory, 1 Cyclotron Rd, Berkeley, CA 94720 USA; 7 UCLouvain, Institut de la Matière Condensée et des Nanosciences (IMCN), Chemin des Étoiles 8, Louvain-la-Neuve, 1348 Belgium; 8 Department of Physics, King’s College London, London, United Kingdom; 9 Department of Materials Science and Engineering, Northwestern University, Evanston, IL 60208 USA; 10 Institute of Biotechnology, Life Science Center, Vilnius University, Saulėtekio av. 7, LT-10257 Vilnius, Lithuania; 11 Institute of Computer Science, Faculty of Mathematics and Informatics, Vilnius University, Naugarduko g. 24, LT-03225 Vilnius, Lithuania; 12 Fritz-Haber-Institut der Max-Planck-Gesellschaft, Faradayweg 4-6, 14195 Berlin, Germany; 13 Center for Autonomous Materials Design, Duke University, Durham, NC 27708 USA; 14 Department of Mechanical Engineering and Materials Science, Duke University, Durham, NC 27708 USA; 15 Humboldt-Universität zu Berlin, Institut für Physik and IRIS Adlershof, 12489 Berlin, Germany; 16 Laboratory of Molecular Simulation (LSMO), École Polytechnique Fédérale de Lausanne, 1951 Sion, Switzerland; 17 National Research Council-Istituto Officina dei Materiali (CNR-IOM), 34136 Trieste, Italy; 18 Karlsruhe Institute of Technology (KIT), Hermann-von-Helmholtz-Platz 1, 76344 Eggenstein-Leopoldshafen, Germany; 19 Materials Measurement Laboratory, National Institute of Standards and Technology, Gaithersburg, MD 20899 USA; 20 Ansys, 300 Rustat House, 62 Clifton Rd, Cambridge, CB1 7EG United Kingdom; 21 Dutch Institute for Fundamental Energy Research (DIFFER), De Zaale 20, 5612 AJ Eindhoven, The Netherlands; 22 Department of Physics and Science of Advanced Materials Program, Central Michigan University, Mount Pleasant, Michigan 48859 USA; 23 Argonne National Laboratory, Lemont, IL 60439 USA; 24 Thayer School of Engineering, Dartmouth College, Hanover, NH 03755 USA; 25 Toyota Research Institute (TRI), Los Altos, California, 94022 USA; 26 Department of Computer Science, University of Copenhagen, Universitetsparken 1, 2100 Copenhagen, Denmark; 27 John A. Paulson School of Engineering and Applied Sciences, Harvard University, Cambridge, Massachusetts 02138 USA; 28 Robert Bosch LLC, Research and Technology Center North America, 255 Main St, Cambridge, Massachusetts 02142 USA; 29 School of Metallurgy and Materials, University of Birmingham, Edgbaston, Birmingham, B15 2TT United Kingdom; 30 Departments of Materials and Physics, and the Thomas Young Centre, Imperial College London, Exhibition Road, London, SW7 2AZ United Kingdom; 31 Department of Material Science and Engineering, Hearst Mining Memorial Building, UC Berkeley, Berkeley, CA 94720 USA; 32 Computer Network Information Center, Chinese Academy of Sciences, Beijing, China; 33 University of Chinese Academy of Sciences, Beijing, China

**Keywords:** Condensed-matter physics, Theory and computation, Condensed-matter physics, Computational science

## Abstract

The Open Databases Integration for Materials Design (OPTIMADE) consortium has designed a universal application programming interface (API) to make materials databases accessible and interoperable. We outline the first stable release of the specification, v1.0, which is already supported by many leading databases and several software packages. We illustrate the advantages of the OPTIMADE API through worked examples on each of the public materials databases that support the full API specification.

## Introduction

Data has become a crucial resource in many scientific fields, and materials science is no exception. Experimental data has long been meticulously curated in handbooks and databases, with the first edition of Landolt-Börnstein^1^ being published in 1883. Nowadays, various commercial and non-commercial experimental databases, such as the Inorganic Crystal Structure Database (ICSD)^2^, are widely used throughout the field.

High-throughput electronic structure calculations, themselves enabled by algorithmic improvements and growing computational resources, have significantly increased the availability of useful data from computational simulations of materials. Since the pioneering work of Ceder *et al*.^3^, a large number of high-throughput first-principles studies have been reported in the literature (for a review, see ref. ^4^), with results typically collated in databases. This explosion in the amount of available data has kick-started a new paradigm of data-driven materials science^5^, creating opportunities for concurrent, automated materials design, boosted by databases that can be queried by humans and machines via an application programming interface (API)^6–9^.

As materials databases differ in fidelity and focus across material classes and properties, it is extremely beneficial to be able to liberate and unify data from multiple sources. However, retrieving data from multiple databases is difficult as each database has its own specialized, and sometimes esoteric, API that governs data access patterns, querying and the representation of the underlying data. Moreover, as the APIs of individual databases inevitably evolve, existing clients must also evolve; a significant maintenance effort is required to translate the responses from the new API to the representation of the client.

Motivated by these considerations, providers of several materials databases united to design and implement an API specification that enables seamless access and interoperability across materials databases. The effort started at the workshop “Open Databases Integration for Materials Design”, held at the Lorentz Center in Leiden, Netherlands in October 2016, and continued at followup workshops held at CECAM in Lausanne, Switzerland in June 2018, June 2019, and June 2020. The result is the OPTIMADE specification (v1.0)^10^; OPTIMADE defines a RESTful API that is queried with URLs, with responses adhering to the JSON:API specification^11^. Specification development adheres to Semantic Versioning^12^ to avoid surprises and enable backwards-compatibility where possible, without impeding further development. By extracting the technical and scientific commonalities from existing APIs, the OPTIMADE API has been designed so that it can be implemented across a broad range of materials domains, database back-ends and sizes.

In this paper, we first review the query format of existing databases to motivate the design and construction of the OPTIMADE API specification. We then illustrate the use of the API with a set of worked examples; databases that already fully support the OPTIMADE API are enumerated alongside their results for representative queries in Table 1. We further highlight libraries that could accelerate uptake and assist materials data curators to support the OPTIMADE API format. Finally, we discuss future prospects and ongoing development of the OPTIMADE API.

**Table 1:** Materials databases with active OPTIMADE API implementations and the number of entries they return for the filters presented in this paper.

Provider	N_1_	N_2_	N_3_
AFLOW^26,27^	700,192	62,293	382,554
Crystallography Open Database (COD)^28,29^	416,314	3,896	32,420
Theoretical Crystallography Open Database (TCOD)^19^	2,631	296	660
Materials Cloud^9,16,17^	886,518	801,382	103,075
Materials Project^21,24,30,31^	27,309	3,545	10,501
Novel Materials Discovery Laboratory (NOMAD)^22,32^	3,359,594	532,123	1,611,302
Open Database of Xtals (odbx)^33^	55	54	0
Open Materials Database (*omdb*)^34^	58,718	690	7,428
Open Quantum Materials Database (OQMD)^35^	153,113	11,011	70,252

The OPTIMADE website provides an up-to-date record of the implementation status of the databases (https://www.optimade.org/providers-dashboard/). AFLOW, Materials Project, odbx, *omdb*, and OQMD comprise computational materials data generated using database-specific workflows^21,27,33–35^. For the purposes of this table, Materials Cloud results were aggregated across all provided sub-databases. COD and TCOD comprise experimental and theoretical crystal structure data extracted from the literature. Materials Cloud comprises materials data from computational workflows; sub-databases group data by research project and can be contributed by users^9^. NOMAD aggregates computational data from multiple sources including from several of the repositories listed here.

## Current Generation of Materials Database APIs

Materials databases are a veritable treasure trove of information, but they only become useful once a human, or machine, can access them. In this section we review the current range of APIs used by various databases to enable access to an example compound, SiO_2_, which serves to highlight the variation of APIs that a user must navigate in order to make use of multiple materials databases. We then demonstrate the universal nature of the OPTIMADE API that permits seamless access to all materials databases that support it.

We first compare and contrast the APIs that must be used to request records on an exemplar system, SiO_2_, from three different databases: AFLOW, the Materials Project, and the Crystallographic Open Database (COD). All three queried databases support requests using a representational state transfer through a web service (RESTful), at the following URLs:

AFLOW      http://aflow.org/API/aflux/?species(Si,O),nspecies(2)

Materials Project    https://www.materialsproject.org/rest/v2/materials/SiO2/vasp/structure

COD       https://www.crystallography.net/cod/result.php?formula=O2%20Si

Note that the Materials Project requires the user to supply an API key (http://materialsproject.org/open) preferably specified in the X-API-KEY HTTP header.

The three APIs vary syntactically (in format), taxonomically (having different names for terms), and semantically (in the conflicting definitions of chemical formula as an intensive or extensive property). AFLOW returns all structures with both Si and O present, whereas both the Materials Project and COD deliver any structure with a formula unit of SiO_2_. The wide range of query formats that will deliver non-overlapping structures significantly complicates access to all available data for SiO_2_, without even considering the differing representations of the structures returned.

The inconsistent format of the query is further complicated by the difficulty of accessing other structures with the SiO_2_ formula. Focusing on just AFLOW, two possible queries that users more familiar with the other APIs might attempt are

http://aflow.org/API/aflux/?compound(SiO2)

which returns no response;

http://aflow.org/API/aflux/?compound(O2Si1)

now lists the elements in alphabetical order as required by AFLOW, and includes the “1” after element symbols, so that “SiO2” becomes “O2Si1”. This returns entries where the unit cell is SiO_2_, but does not return Si_2_O_4_ or simulation cells containing more formula units.

The exemplar http://aflow.org/API/aflux/?species(Si,O),nspecies(2) returns all entries with at least one Si and an O, so while the response includes the SiO_2_ phases of interest, it may also contain other stoichiometries.

The distinctions between the request format for each database require the user to become an expert in many different APIs. This again emphasises the need for a single well-designed and standardized API to access all materials databases, which is the aim of the OPTIMADE API.

## The OPTIMADE API

The OPTIMADE API provides a holistic standard for serving and accessing the information in compatible materials databases. To retrieve information about materials from a particular database, the user submits a request via a URL. Each database provider will have published a base URL that serves the OPTIMADE API, for example https://example.com/optimade/. The same URL path, across different OPTIMADE API implementations, allows uniform access to the underlying databases. Both human-readable and machine-readable versions of the OPTIMADE API specification are available online with releases archived at Zenodo^10^. The specification is also registered as an API standard on FAIRsharing.org^13^.

### Design philosophy

The OPTIMADE specification strives to enable materials information to be filtered and retrieved in a straightforward and intuitive manner. The three queries from the previous section can each be performed on a standardised, versioned endpoint (/v1/structures) that enables access to a structures entry resource type that consists of many well-defined attributes. The specification then defines a grammar for filtering entries against these attributes, allowing the previous SiO_2_ f ilter example to be expressed in a common way (?filter=chemical_formula_reduced="O2Si"). Altogether, the universal OPTIMADE URL, where only the implementation URL changes, becomes:

<optimade_implementation_url>/v1/structures?filter=chemical_formula_reduced="O2Si"

The OPTIMADE specification also aims to be flexible to many different underlying data representations, and thus there are very few properties that are mandatory. Instead of enforcing an exhaustive set of property definitions, individual OPTIMADE implementations can describe the data they serve via /info endpoints for each entry type. These introspective endpoints allow clients to adapt to the particular implementation for an underlying database, and allow providers to disseminate properties beyond the simple structural and chemical information standardized by the specification. To avoid naming collisions, each provider-specific property name must be prefixed by a provider-specific token, itself bookended by underscores (_). The property custom_property from an example provider with assigned prefix exmpl would be expressed as _exmpl_custom_property: e.g., _tcod_a for the lattice constant, *a*, in TCOD, or _aflow_spacegroup_relax for the space group of the relaxed structure in AFLOW.

### Implementation discovery

The list of implementations confirmed and tested in this paper to support the OPTIMADE API is shown in Table 1. They are all publicly accessible, providing users with open access to large international repositories of computational and experimental materials science data.

The OPTIMADE consortium provides an open, federated list of implementations (https://providers.optimade.org). It is considered to be a catalogue of currently available and/or known public OPTIMADE implementations. New implementations are welcome and can register themselves via a pull request on GitHub (https://github.com/Materials-Consortia/providers).

The requirements for appearing in the above providers list are very loose. Some databases listed in the catalogue are signalling the intent of future implementations, while others only have partial implementations of the OPTIMADE API, including JARVIS (https://jarvis.nist.gov/optimade)^14^ and MatCloud (https://matcloud.com.cn)^15^. Some software frameworks, such as AiiDA^16–18^, also enable users to access their personal data through an OPTIMADE API, and therefore have a dedicated provider-specific ID, but no single official OPTIMADE implementation base URL.

The OPTIMADE API also specifies an endpoint for semi-automated cross-provider discovery. The /links endpoint serves links resources that may refer to either provider internal (child, root) or external (external, providers) resources based on the link_type attribute. To avoid being overly restrictive, it is at the provider’s discretion whether they serve a list of known providers; however, this provides a mechanism for scalable and decentralised discovery of new implementations beyond the federated provider list.

## Worked Example

To illustrate the effective use of the OPTIMADE API we now provide a worked example of querying structures. We explore materials containing Group 14 elements (the carbon family), starting with a general search before drilling down to specific materials. The Group 14 elements are of particular interest as their atomic orbitals regularly hybridise, enabling a variety of bonding with differing geometries. The hybridised orbitals enable these elements to form the backbone of a wide range of compounds, both inorganic and organic, that underpin plastics, drugs, and semiconductors. Group 14 therefore forms both a diverse and important family of compounds that heavily populates databases, so are an ideal case study to demonstrate the OPTIMADE API.

### Common features of the response

Whilst our previous exploration of the Group 14 compounds considered only SiO_2_, the flexibility of the OPTIMADE API allows us to start with a search over all materials in Group 14, comprising carbon (C), silicon (Si), germanium (Ge), tin (Sn), and lead (Pb). We start with a simple API call that searches for all materials that contain at least one element in Group 14:

/v1/structures?filter=elements HAS ANY "C", "Si", "Ge", "Sn", "Pb"

This string can be appended to the base URL of any of the available implementations, to gather results in a standardised form. The base URL can be found on the providers dashboard (https://www.optimade.org/providers-dashboard).

As an example, this query is run through the Theoretical Crystallography Open Database (TCOD)^19^ with the following URL:

https://www.crystallography.net/tcod/optimade/v1/structures?filter=elements+HAS+ANY+"C","Si","Ge","Sn","Pb"

The JSON response is summarized in Boxes 1 through 5, where some lines have been omitted for brevity; the full response is given in Supplementary File 1.

The first tranche of the JSON response comprises the “data” field that contains a list of entries returned for the query; a truncated version of this field is shown in Box 1, displaying a few salient properties of just one of the ten entries from the full response. The response for a particular material entry comprises multiple sections:

attributes. Box 1 shows the physical properties of the material comprising both mandatory information such as elements and lattice_vectors, as well as optional, additional database-specific information prefixed with the database name (e.g., _tcod_, here used to provide lattice parameters). This ensures that all databases return the most important and common information in a standardized format, as well as allowing them to include additional database-specific data. Importantly, the OPTIMADE specification provides a standardized way for database implementations to be self-documenting, via introspective /info endpoints. We see in the elements section that here we have returned a material comprising the element of interest, Sn, as well as O and Ta.

id and links. Box 2 shows the unique ID for the entry within the database, and a self-link to the database-specific representation/rendering of the entry (in this case, a link to a Crystallographic Information File^20^).

relationships. The relationships section in Box 3 links the user to other entries in the database and beyond, here the bibliographic references.

The additional nine materials not shown here all comprised of compounds containing either C, Si, Ge, Sn, or Pb, supplemented by a variety of other elements. The foot of the response contains information about the underlying database, comprised of three sections:

links. The response returned the first ten (i.e., the default page limit) entries that matched the query, however, more materials are available within the database. Box 4 shows the JSON:API-compliant pagination links to the current and next page of results for this query, as well as relevant external links.

meta. Box 5 provides metadata associated with the request, such as number of results, the details of the database provider, the implementation and the representation and timestamp of the submitted query.

A benefit of the OPTIMADE API is that the structure of the response is common to all materials databases. The responses differ only in the optional and database-specific information prefixed with the database name (here _tcod_). Table 1 lists several materials databases that have active OPTIMADE API implementations, and the large number of results (**N**_**1**_) that they return for this particular filter.

Box 1 An excerpt of the JSON response showing the material attributes for one of the returned structures entries.

Box 2 An excerpt of the JSON response showing the top-level entry ID and a self-link.

Box 3 An excerpt JSON data response for links to other entries related to this structure in the database (here, a bibliographic references entry).

Box 4 An excerpt of the JSON response for navigable pagination links to additional conformant materials that match the query.

Box 5 An excerpt of the JSON response showing the metadata returned for this request.

### Database filtering

Requesting the example filter above from the TCOD database returns 2,631 materials entries, but the same filter could return millions (see Table 1). For some requests the volume of the materials data could become unmanageable so the specification allows for the use of several pagination methods laid out by JSON:API^11^. These approaches all provide a link to the “next” page of data:

/v1/structures?page_limit=10&page_offset=10&filter=elements HAS ANY "C", "Si", "Ge", "Sn", "Pb"

with parameter page_offset = 10 to allow the user to select which page to enter, and the parameter page_limit = 10 to control the number of materials returned per individual request.

The most useful way to explore an OPTIMADE database is to apply a filter; the specification mandates that several relevant properties must be queryable. For example, we can perform a more focused search for materials comprising at least one element in Group 14, and a maximum of two elements (a binary material), with the filter

/v1/structures?filter=elements HAS ANY "C", "Si", "Ge", "Sn", "Pb" AND nelements=2

This query returns 296 materials from the TCOD database, with the response summarized in Box 6, where some lines have been omitted for brevity and the full response is given in the Supplementary File 2. The number of matching entries (**N**_**2**_) across all implementations for this filter are shown in Table 1.

We can now see that the first structure, and indeed all structures, returned are comprised of at least one element in Group 14, here Ge, and a maximum of one other element (a binary material), here O. Additional filters can be chained to further refine the materials returned, or to construct more complex queries. For example, ternary structures that contain at least one of the elements C, Si, Ge, or Sn, but do not contain Pb (e.g., for applications where Pb toxicity would be a concern), can be retrieved using the filter

/v1/structures?filter=elements HAS ANY "C", "Si", "Ge", "Sn" AND NOT elements HAS "Pb" AND elements LENGTH 3

The number of entries matching this filter are denoted as **N**_**3**_ in Table 1.

These simple examples demonstrate how useful chemical queries are expressible with the OPTIMADE API, allowing users to refine their queries and to suit their specific application. Further functionality of the OPTIMADE API can be found in the specification^10^.

Box 6 The truncated JSON response for the more focused search, showing the elements in the first material returned.

## Related Libraries

The wider usage of the OPTIMADE API is a key goal for the consortium; to this end, several open source libraries have been developed to help users of the OPTIMADE API (either implementation developers, or client end-users), of which three are introduced below. The first two libraries offer tools that aid the implementation of the API for materials database developers, with the first also containing tools to construct and validate queries, while the third library is intended for end users of OPTIMADE-compliant APIs.

### optimade-python-tools

optimade-python-tools is an open source Python package available on GitHub (https://github.com/Materials-Consortia/optimade-python-tools). The package contains a complete set of tools for implementing an OPTIMADE-compliant API, as well as several utilities that can be used by client code. The package is listed on the Python Package Index (PyPI) as optimade (https://pypi.org/project/optimade). Current (v0.14) functionality of the package includes:

- pydantic (https://github.com/samuelcolvin/pydantic) data and validation models for all objects defined in the OPTIMADE specification that can be used in server or client code;
- an extensible reference server implementation leveraging pydantic and FastAPI (https://github.com/tiangolo/fastapi). This reference server forms the basis of the OPTIMADE implementations for the Materials Project^21^, NOMAD^22^, Materials Cloud^9^ and odbx (https://odbx.science) providers. These tools are also used to generate a machine-readable OpenAPI version^23^ of the OPTIMADE specification;
- a validator for OPTIMADE implementations, available standalone with a command line interface (CLI) or as a GitHub action (https://github.com/Materials-Consortia/optimade-validator-action). This validator is run against the federated list of OPTIMADE providers every day, with a live dashboard indicating compliance with the specification at https://www.optimade.org/providers-dashboard/, the source code for which can be found on GitHub (https://github.com/Materials-Consortia/providers-dashboard);
- a Python parser for the OPTIMADE filter language written with the Lark parsing library (https://github.com/lark-parser/lark);
- filter transformers from the abstract syntax tree to queries to popular database back-ends. Currently, the supported back-ends are MongoDB (via pymongo) (https://github.com/mongodb) and Elasticsearch (https://github.com/elastic/elasticsearch);
- adapter classes to interface with other popular libraries, namely pymatgen^24^, ASE^25^, AiiDA^16–18^ and JARVIS^14^ as well as converting OPTIMADE structures to the CIF^20^, XYZ, and more domain standardised file formats.

## OPTIMADE::Filter

OPTIMADE::Filter is a Perl library for the syntactical analysis of the OPTIMADE filter language. Apart from the construction of abstract syntax trees, the library can translate simple filter strings to SQL queries. The Git repository with the source code is publicly available on GitHub (https://github.com/Materials-Consortia/OPTIMADE-Filter).

### pymatgen optimade module

pymatgen^24^ (https://pymatgen.org) is a Python library for materials science. A user-friendly OptimadeRester client has been added to a new OPTIMADE module within pymatgen to provide a way to query OPTIMADE structure resources in a way familiar to existing users of pymatgen and the Materials Project API. The Git repository with the source code is publicly available on GitHub (https://github.com/materialsproject/pymatgen).

## Summary

The latest OPTIMADE API specification v1.0^10^ offers holistic access to many leading crystal structure databases, namely: AFLOW, COD, TCOD, Materials Cloud, Materials Project, NOMAD, odbx, Open Materials Database (*omdb*), and OQMD. Open client implementations are also available (https://optimade.science, https://materialscloud.org/optimadeclient) that enable aggregated searches over many databases as well as user-friendly graphical widgets that can create an OPTIMADE filter to empower the user with even easier access to data. OPTIMADE provides researchers easy access to over 10000000 results for different materials, providing benchmarking opportunities and offering a huge opportunity for high-throughput screening and machine learning studies. The ability of the OPTIMADE API to search databases, expose links between databases, and deliver standardized results makes it well-positioned to significantly enhance the impact and permeability of pre-existing data silos. This should empower researchers to scan through new and unexpected material families, and train models from all available data that can understand deep correlations.

The OPTIMADE API is flexible and will be extended to more use cases going forward. The development and adoption of the OPTIMADE API relies on the involvement of a large number of scientists, so contributions from the community are strongly encouraged, and questions on development, registration of a provider, or usage can be directed to the web forum (https://matsci.org/optimade) or mailing list (dev@optimade.org). Proposed developments include the standardization of more filterable materials properties, the integration of molecular dynamics simulations and of experimental results, and extensions beyond electronic-structure calculations. The future development of APIs, including OPTIMADE, should herald an era of effective use of big, open data in materials science.

## Supplementary information

Supplementary File 1

Supplementary File 2

## Data Availability

The OPTIMADE specification is developed openly on GitHub with releases archived on Zenodo^10^. Version 1.0.0 was released on 1 July 2020 and is licensed under Creative Commons Attribution 4.0 International (CC-BY 4.0).

## Code availability

All associated code is hosted under the Materials-Consortia organisation on GitHub (https://github.com/Materials-Consortia).
